# Precise tracking of tip-induced structural variation at the single-chemical-bond limit

**DOI:** 10.1038/s41377-022-01055-5

**Published:** 2023-01-11

**Authors:** Sayantan Mahapatra, Nan Jiang

**Affiliations:** Department of Chemistry, University of Illinois Chicago, Chicago, IL 60607 USA

**Keywords:** Raman spectroscopy, Nanophotonics and plasmonics

## Abstract

Sub-nanometer-resolved TERS provides a systematic way for investigating tip-molecule interaction and molecular motions, enabling a promising approach to examine on-surface reaction mechanisms and catalysis at the microscopic level.

## Abstract (web summary)

Tip-molecule interactions and molecular motions such as molecular tilting and hopping process were explored via sub-nanometer-resolved tip-enhanced Raman Spectroscopy with single-chemical-bond limit.

## Graphical abstract

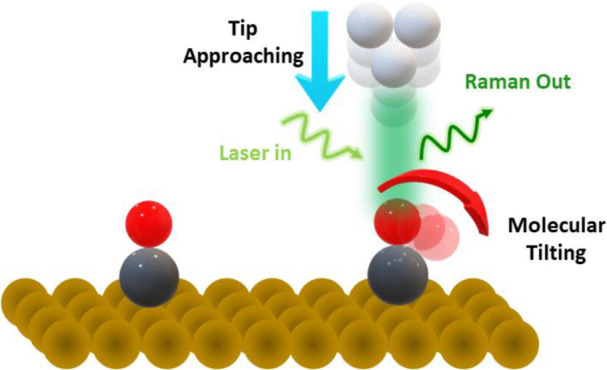

The realization of optical spectroscopy at the atomic scale is one of the utmost goals in the nano-optics research field, providing an exceptional ability to investigate the light-matter interaction at the sub-nanometer scale. At these nano-dimensions, quantum phenomena dictate the intricate details of atoms and molecules, thereby calling for a technique to interrogate the structure with suitable spatial resolution. In that sense, scanning tunneling microscopy (STM) provides real-space images of individual atoms and molecules by detecting the tunneling current between the tip and the sample. However, it notably lacks chemical sensitivity, making life extremely difficult to characterize the surface superstructures by STM alone. Instead, STM combined with Raman spectroscopic technique, i.e., tip-enhanced Raman spectroscopy (TERS), offers researchers an efficient way to interrogate the sub-nanoscale photochemical and photophysical processes^1^. Over the last decade, angstrom-scale chemical analysis has matured into a platform that validates the investigation of fundamental properties of individual molecules at the atomic scale^2–8^.

For TERS, one of the key factors is the utilization of a plasmonically active STM tip (made of silver or gold). The confinement of light at the STM tip apex provides a localized and intense electromagnetic (EM) field and enhances the chemical analysis methods (such as Raman scattering), even to a point where it is feasible to detect a single chemical bond^9^. As the plasmonic enhancement relies inversely on the confinement volume, the sub-nanoscale chemical investigation requires squeezing the EM field inside a tiny cavity (typically known as “pico-cavity”). The utilization of ultrahigh vacuum (UHV) and cryogenic temperature provides the finest control of such a small volume with sub-nanometer precision. In addition, the nano-confined light can be further concentrated by lowering the tip-sample nano-gap^10^. However, at these small tip-sample gap distances, the delicate physico-chemical details of how the tip interacts with the surface-adsorbed molecule are still a matter of debate^11,12^. It is particularly intriguing, as the tip-molecule interaction could significantly influence the chemical structure and molecular movements.

Now, writing in this issue of Light: Advanced Manufacturing, Xiao-Ru Dong and colleagues at the University of Science and Technology of China investigate the tip-molecule interaction and the structural variations of an individual surface-adsorbed molecule via cryogenic sub-nanometer-resolved TERS^13^. Utilizing a single upstanding carbon monoxide (CO) molecule adsorbed over Cu(100) surface as a model template, the authors successfully shed light on various tip-induced chemical processes such as bond weakening, tilting, and hopping for a single molecule on the surface at the single-chemical-bond limit.

In the work presented here, the authors tracked the tip-induced variation in the C–O stretching mode for an individual surface-adsorbed CO molecule. With approaching the Ag tip towards a CO molecule, a continuous red-shift was observed associated with the tip-induced weakening of the C–O bond, which is a direct consequence of the force field applied by the STM tip. Furthermore, high-resolution TERS spatial mapping (~5.6 Å spatial resolution) showcased the tilting phenomena of an individual CO molecule, ultimately leading to lateral hopping upon further tip approaching. These impressive results demonstrate high-resolution TERS as an appealing approach to investigating tip-molecule interactions and the tip-induced on-surface molecular motion at the single-chemical-bond limit.

The interplay between one surface-adsorbed molecule and the force field of the STM tip provides precious insights into the single-molecule structure manipulation process. As presented in this study, the tip-molecule interactions can be categorized into two distinct regimes, depending on the tip-substrate gap distances. For the larger gap distance (i.e., >3.90 Å), the bond weakening is attributed to the attractive van-der-Waals interactions between the tip and the molecule (Fig. 1). However, as the tip approaches below 3.90 Å, the Pauli repulsive force dominates, introducing tilting in the CO molecule. Such a tilting also provides more charge transfer to the antibonding orbital of CO, leading to the further weakening of the C–O bond. Furthermore, below 1.46 Å, the molecule starts to diffuse to the nearest adsorption site (Fig. 1). By carefully tracking the C–O stretching mode and theoretical simulations, the authors obtained critical details of the molecular movements and hopping mechanism, leading a two-step hopping process of the CO molecule to the neighboring Cu atom via the bridge site.

**Fig. 1:**
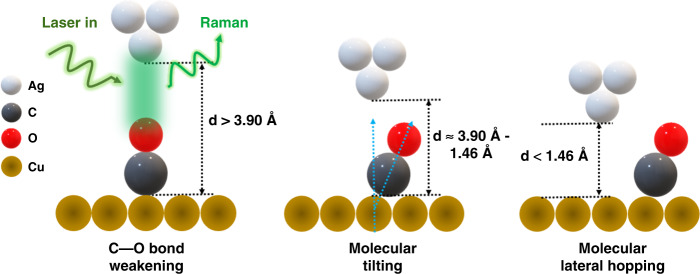
Schematics of high-resolution TERS and characterization of tip-molecule interactions at the single-chemical-bond limit.

The successful realization of tip-molecule interaction phenomena and mechanisms at the microscopic scale demonstrates the remarkable accomplishment of angstrom-scale TERS, making it suitable for surface reaction mechanisms and catalysis at the single-chemical-bond level. With the recent advancements in the nano-confinement of light for optical spectroscopy and chemical reactions, we can expect that this fundamental work will promote further fascinating research with the tip-assisted molecular motions and structural change, pushing its applicability to an unprecedented level. This will also unlock the door to many other applications where tip-molecule interactions remain extremely critical, such as site-selective plasmon-induced chemical reactions^14^, single-molecule nano-engineering^15^, and single-molecule electron-transport processes^16^.
